# Galectin-7 is epigenetically-regulated tumor suppressor in gastric cancer

**DOI:** 10.18632/oncotarget.1219

**Published:** 2013-08-20

**Authors:** Seok-Jun Kim, Jung-Ah Hwang, Jae Y. Ro, Yeon-Su Lee, Kyung-Hee Chun

**Affiliations:** ^1^ Department of Biochemistry & Molecular Biology, Yonsei University College of Medicine, 50 Yonsei-ro, Seodaemun-gu, Seoul, Republic of Korea; ^2^ Cancer Genomics Branch, Division of Fusion Technology, National Cancer Center, 323 Ilsan-ro, Ilsandong-gu, Goyang-si, Gyeonggi-do, Republic of Korea; ^3^ The Methodist Hospital, Department of Pathology and Genomic Medicine, Weill Medical College of Cornell University, Houston, TX, USA

**Keywords:** Galectin-7, Gastric cancer, DNA hypermethylation, Epigenetic mechanisms

## Abstract

Gastric cancer is the second leading cause of cancer death and remains a major clinical challenge due to poor prognosis and limited treatment options. Therefore, the basic mechanisms underlying gastric tumorigenesis deserve investigation. Although regulation of the galactoside-binding lectin galectin-7 in cancer has been studied, its role in tumor formation and progression remains controversial. In this study, we investigated galectin-7 expression and its role in gastric cancer. Immunohistochemical staining using a tissue microarray of gastric cancer patients revealed significantly low expression levels of galectin-7 in malignant tissues compared with matched normal tissues, and decreased expression of galectin-7 in malignant tissues was associated with advanced TMN stage disease (p =0.034). Importantly, low expression of galectin-7 in normal tissues was associated with a poor survival rate (p =0.0561). Over-expression of galectin-7 in AGS gastric adenocarcinoma cells suppressed cell proliferation, migration, and invasion, whereas ablation of galectin-7 in KATO III gastric carcinoma cells reversed these properties. AGS cells that overexpressed galectin-7 could not form gastric tumors in xenografted mice. More than 70% hypermethylation was observed in 7 of 9 gastric cancer cell lines tested and 5-aza-cytidine treatment lowered galectin-7 expression by reducing methylation in 24 cancer cell lines from five different organ origins. We analyzed CpG islands in the galectin-7 genomic region and detected hypermethylation at +1566bp of exon 2, the predicted p53 binding region. DNA hypermethylation of this region was also detected in gastric cancer tissues from 20 patients. Taken together, our data indicate that galectin-7 has a tumor suppressive function, and that the gene is epigenetically modified by DNA methylation and significantly down-regulated in gastric cancer. Further study of galectin-7 regulation may lead to improved gastric cancer diagnosis and therapy.

## INTRODUCTION

Galectin-7 is a member of a family of proteins with affinity for β-galactosidase–containing oligosaccharides. Galactin-7 contains one carbohydrate recognition domain (CRD) in its biological structure and functions as a homodimer [1-2]. In contrast to other galectins such as galectin-1 and −3, the function of galectin-7 is still largely unknown. It is known to interact with a wide range of potential receptors, including non-reducing terminal LacNac residues and internal LacNac oligosaccharide residues, but its carbohydrate binding affinity is weaker than that of galectin-1 and −3 [3]. It has been suggested that the function of galectin-7 may vary according to its cellular localization because the protein is present in the nucleus and the cytoplasm, and also in the cell-to-cell contact region.

Research performed over the last two decades has shown that galectin-7 is associated with the differentiation and development of epithelia, including epithelial cell migration and epidermal wound healing [4-5]. The regulation of apoptosis induction by galectin-7 also has been studied [6-9]. This regulation is thought to involve JNK activation, mitochondrial cytochrome c release, and Bcl2 binding in the mitochondrial membrane. In addition, there are several reports of a role of galectin-7 in cancer development. Altered galectin-7 expression is involved in tumor progression through the regulation of cell proliferation, apoptosis induction, and cell invasion [9-12]. Interestingly, galectin-7 may contribute to the suppression of cancer proliferation in certain tumor types and induce the growth and metastasis of others. For the suppressive function, galectin-7 was identified as a p53-induced gene 1 (PIG1) and was shown to reduce neuroblastoma cell proliferation [10]. Etopic expression of galectin-7 in cancer cells increased their susceptibility to apoptosis and suppressed tumor growth [7, 11]. Both galectin-7 and S100A9 were found to play protective roles in cervical squamous carcinomas [13]. In contrast, stimulatory roles of galectin-7 in cancer development have also been widely demonstrated. Over-expression of galectin-7 modulated the aggressive behavior of lymphoma cells through expression of the metastasis-related gene *MMP-9* [12] and the expression of galectin-7 was increased in rat mammary carcinomas induced by carcinogen [14]. High expression of galectin-7 in breast cancer cells induced their ability to metastasize to lungs and bones, and many breast carcinoma samples contain more than 70% galectin-7– positive cells [15]. Therefore, the precise role of galectin-7 in cancer development is still debated and appears to be tissue specific, which we find fascinating. Moreover, the role of galectin-7 in gastric cancer has not been studied.

In this study, we first determined the differential expression of galectin-7 in gastric cancer cell lines and tissues from gastric cancer patients compared with matched normal tissue. We found that the expression of galectin-7 was down-regulated in malignant tissues from gastric cancer patients and was regulated by DNA methylation of CpG islands in regulatory regions containing a putative p53 binding site. Over-expression of galectin-7 suppressed cell proliferation in p53 wild-type AGS gastric cancer cells. Taken together, these findings suggest that galectin-7 has a suppressive role in gastric cancer and that its expression is regulated by epigenetic mechanisms such as DNA methylation.

## RESULTS

### Galectin-7 expression is down-regulated in malignant tissues from gastric cancer patients relative to matched normal tissue

**Figure 1:**
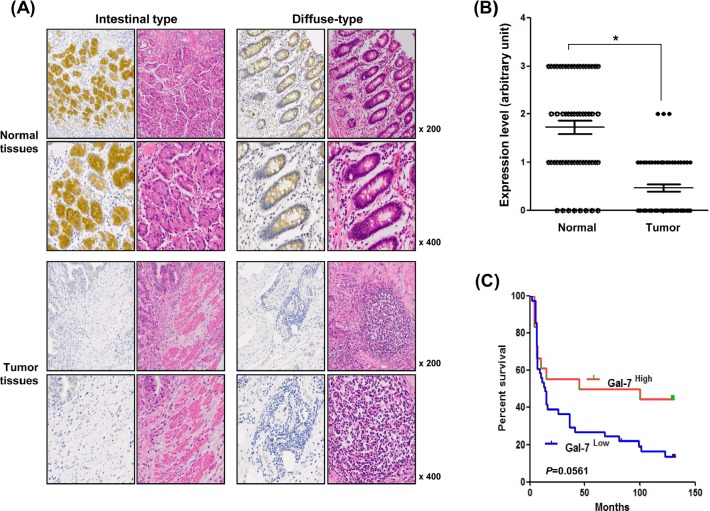
Expression levels of galectin-7 in malignant and normal tissues of 56 gastric cancer patients (A) In situ expression of galectin-7 in normal and tumor tissue from gastric cancer patients of intestinal and diffuse type was detected by immunohistochemical (IHC) analysis and hematoxylin and eosin (H&E) staining (magnification: ×200, ×400). (B) Comparative analysis of galectin-7 expression in normal and tumor tissues of gastric cancer patients based on intensity of galectin-7 staining. *p* values (*, *p*<0.001) were calculated using Student's t-test. (C) Kaplan-Meier analysis of survival curves for patients with gastric cancer (*N* = 44) according to the expression of galectin-7 in normal tissues. The galectin-7 low-expression group showed a much lower survival rate at 125 months compared with the galectin-7 high-expression group.

To determine the expression levels of galectin-7 in gastric cancer patients, we prepared a tissue microarray (TMA) of 44 patients and performed immunohistochemical analysis (Table 1 and Figure 1A). Strong expression was detected in normal tissues from patients with intestinal and diffuse types of gastric cancer and most of the galectin-7 was localized in the cytosol. Expression was notably down-regulated in gastric cancer tissues (Figure 1A). Quantitative analysis of galectin-7 staining confirmed that gastric cancer patients had low or no expression in malignant tissues compared with normal tissues (Figure 1B). As shown in Table 1, we statistically analyzed the expression levels with respect to clinical factors. The protein expression levels of galectin-7 in malignant tissues were significantly decreased in patients with advanced stage disease by T classification (*p*=0.034) (Table 1). Moreover, the survival probability was higher in gastric cancer patients with high galectin-7 expression in normal tissues (*p*=0.0561) (Figure 1C) although this was not statistically significant, possibly due to the limited number of samples. Our data strongly indicate that galectin-7 is down-regulated during gastric cancer progression.

**Table 1:** 

Characteristic	Galectin-7 expression							
	Normal				Tumor			
	Low (N = 30)	High (N =14)	*P* value		Negative (N = 25)	Mild (N = 16)	Moderate (N = 3)	*P* value
**Sex**			0.602					0.206
Male	19	10			18	10	1	
Female	11	4			7	6	2	
**Age (years)**			0.408					0.626
<60	11	7			9	8	1	
≥60	17	7			16	8	2	
**Differentiation**			0.344					0.550
Well	3	1			3	1	0	
Moderately	8	2			4	5	1	
Poorly	12	8			14	4	2	
other	7	3			4	6	0	
**Lauren's classification**		0.669						0.499
Intestinal	13	5			8	9	1	
Mixed	1	0			17	6	2	
Diffuse	16	9			0	1	0	
**T classification**			0.568					0.034*
T1	5	3			3	4	1	
T2	3	2			2	2	1	
T3	14	6			11	8	1	
T4	8	3			9	2	0	
**LN metastasis**			0.895					0.282
Negative	8	4			8	4	0	
Positive	22	10			17	12	3	
**TNM stage**			0.568					0.148
I	5	3			3	4	1	
II	8	6			8	5	1	
III	15	5			12	7	1	
IV	2	0			2	0	0	

### Over-expression of galectin-7 suppressed proliferation, migration, and invasion of AGS gastric cancer cells

**Figure 2:**
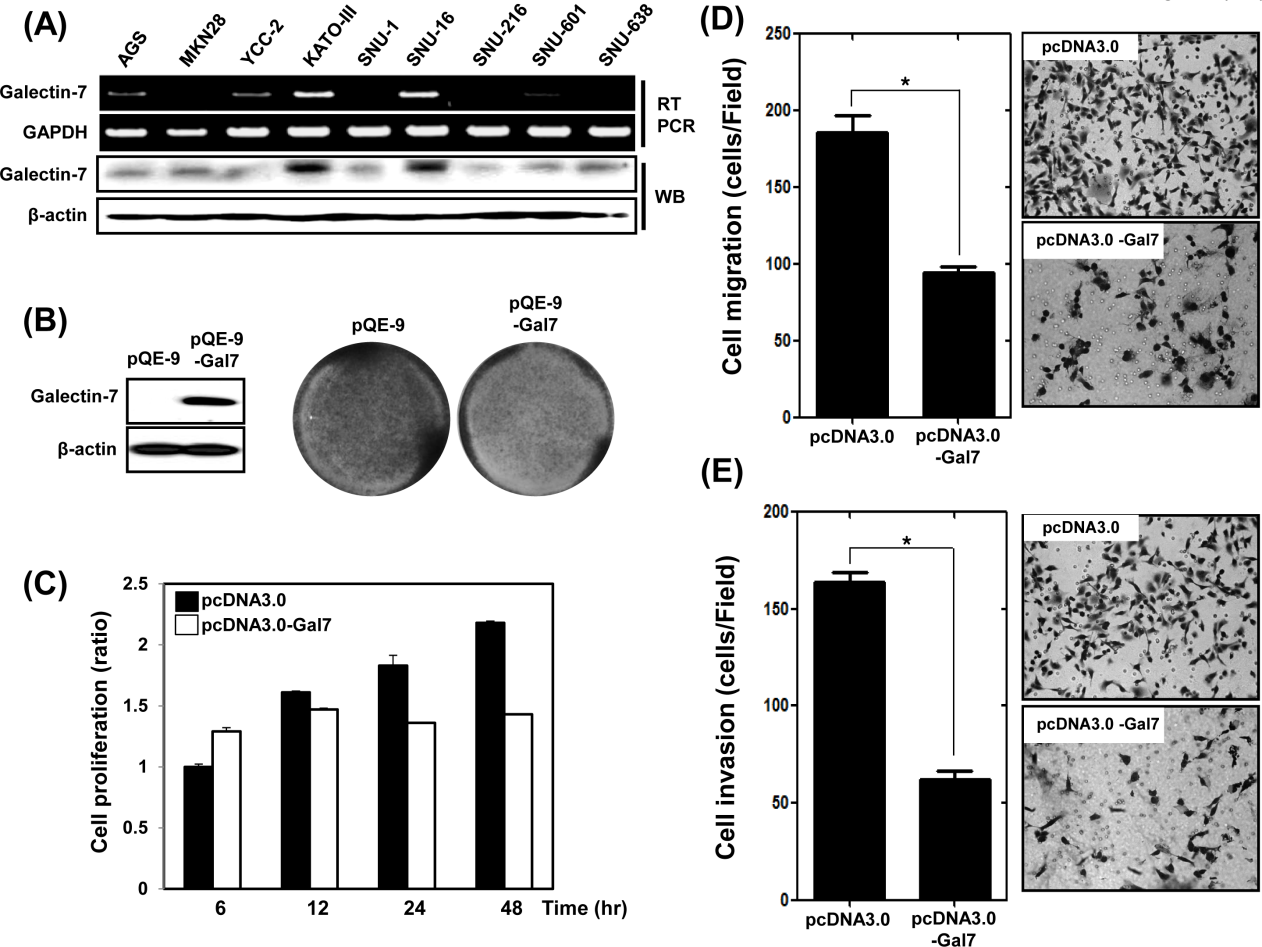
Over-expression of Galectin-7 suppressed proliferation, migration, and invasion of AGS gastric cancer cells (A) mRNA and protein expression of galectin-7 in nine gastric cancer cell lines by RT-PCR and western blot. GAPDH and β-actin were used as loading controls. (B-E) AGS cells were transfected with pQE control vector and pQE-galectin-7 expression vector for 48 h. (B) Western blot analysis of galectin-7 protein expression and crystal violet staining of cells, (C) Cell proliferation measured by WST assay, (D) Cell migration assay, (E) Cell invasion assay. For cell migration and invasion assays the *p* value (* *p*<0.0001 vs. scRNA) was calculated using Student's t-test.

We next investigated the role of galectin-7 in the progression in gastric cancer cells. First, we examined the expression levels of galectin-7 mRNA and protein in nine gastric cancer cell lines (Figure 2A). KATO III and SNU-16 cell lines showed high expression of galectin-7 whereas the other cell lines expressed low levels of galectin-7. We selected AGS gastric cancer cells with low expression of Galectin-7 and transiently transfected them with the expression vector pQE-9-galectin-7 for 48 h. Over-expression of galectin-7 was confirmed by western blotting (Figure 2B). Galectin-7 over-expression inhibited cell proliferation as measured by crystal violet staining (Figure 2B) and WST assay (Figure 2C), and significantly decreased gastric cancer cell migration and invasion, (Figure 2D and E). These findings suggest that galectin-7 functions as a tumor suppressor in gastric cancer.

### Ablation of galectin-7 increased proliferation, migration, and invasion of KATO III gastric cancer cells

**Figure 3:**
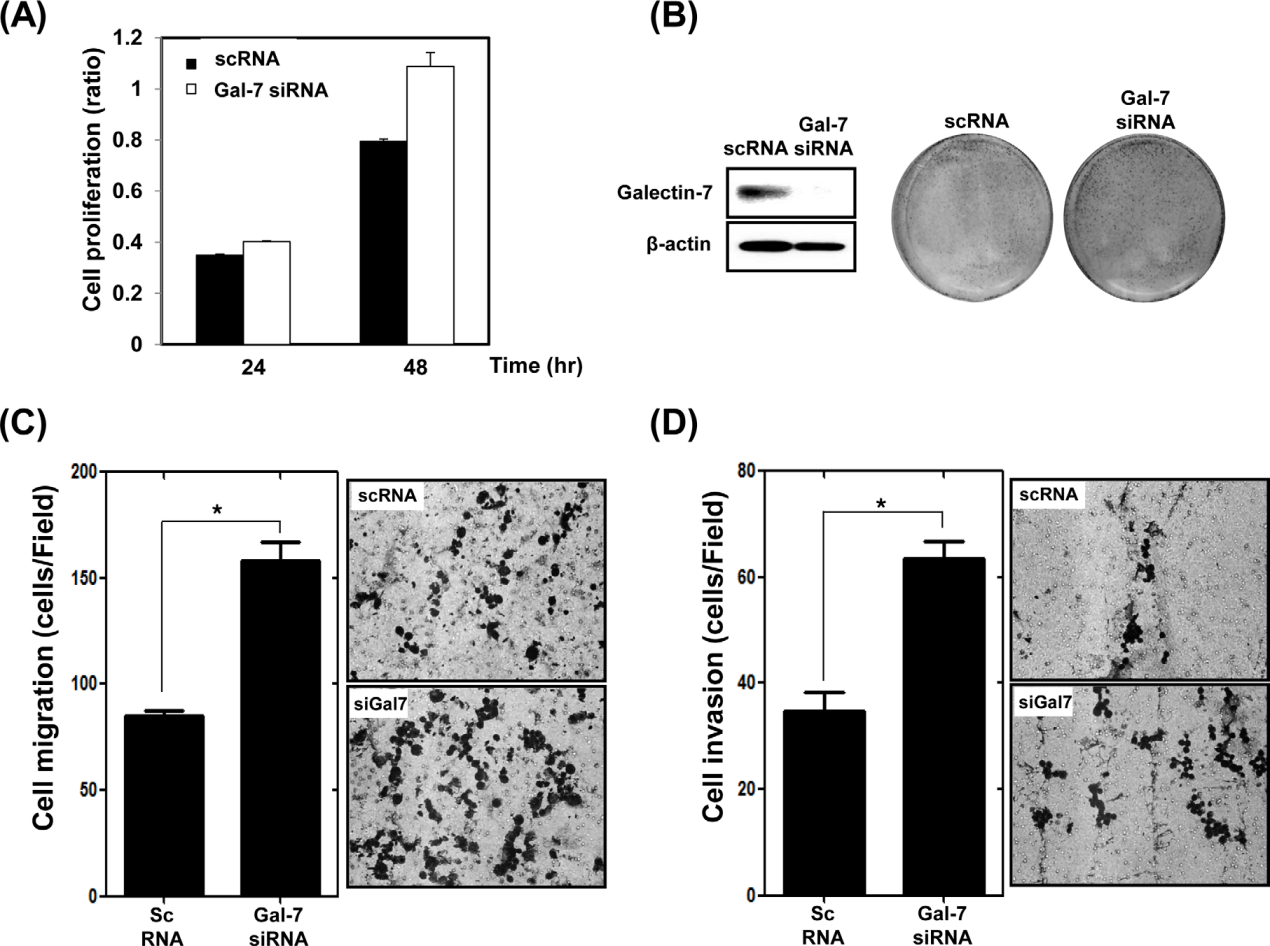
Ablation of Galectin-7 increased proliferation, migration, and invasion of KATO III gastric cancer cells KATO III cells were transfected with scrambled RNA (scRNA) as a negative transfection control and galectin-7 siRNA for 48 h. (A) Cell proliferation using WST assay, (B) detection of galectin-7 protein expression by Western blot and staining with crystal violet, (C) cell migration assay, (D) cell invasion assay. For the cell migration and invasion assays the *p* value (* *p*<0.0001 vs. scRNA) was calculated using Student's t-test.

We also determined the effect of loss of function of galectin-7 in KATO III gastric cancer cells, which have high expression levels of galectin-7. Cells were transfected with scrambled RNA (scRNA) as a negative control and galectin-7 specific siRNA. Ablation of galectin-7 increased cell proliferation (Figure 3A and B), migration (Figure 3C), and invasion (Figure 3D). Those results support the notion that galectin-7 is a tumor suppressor in gastric cancer.

### Effect of in vivo galectin-7 over-expression in gastric cancer cell xenografted mice

**Figure 4:**
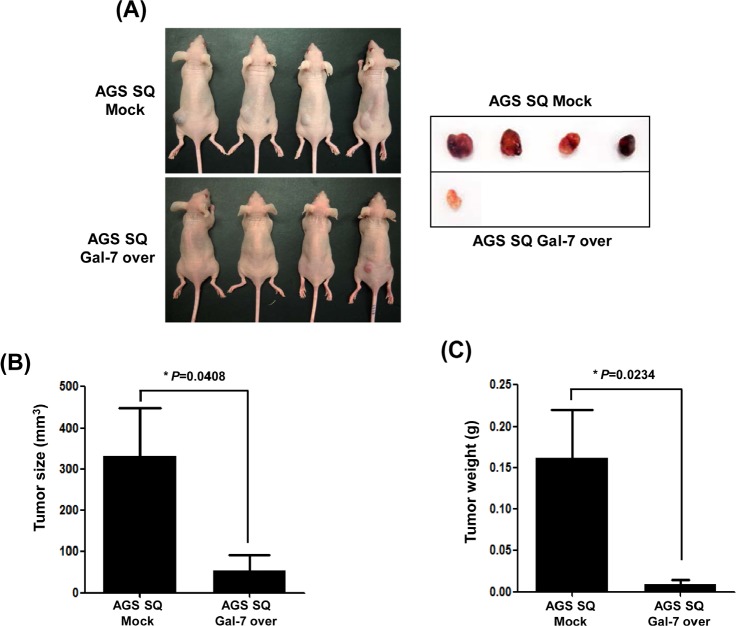
The effect of galectin-7 over-expression on tumor growth of xenografts in nude mice AGS SQ cells (100 μl; 1 × 10^6^ cells) in Matrigel were implanted into Balb/c-nude mice to form subcutaneous xenografts. After 8 weeks the mice were sacrificed, the tumors were photographed (A), and tumor size (B) and weight (C) were measured. The *p* value for tumor size and weight (* *p*=0.048 and 0.0234 vs. Mock groups) was calculated using Student's t-test.

We prepared stable galectin-7 over-expressing AGS cells and inoculated them into nude mice (Figure 4). Although tumors were formed from AGS cells expressing vector control pcDNA 3.0, the galectin-7 over-expressing AGS cells could not form gastric tumors in the xenografted mice (Figure 4). These results strongly suggest that galectin-7 prevents gastric cancer formation and progression.

### DNA hypermethylation in the region of the CpG islands at +1566 bp in exon 2 of the galectin-7 gene in gastric cancers

**Figure 5:**
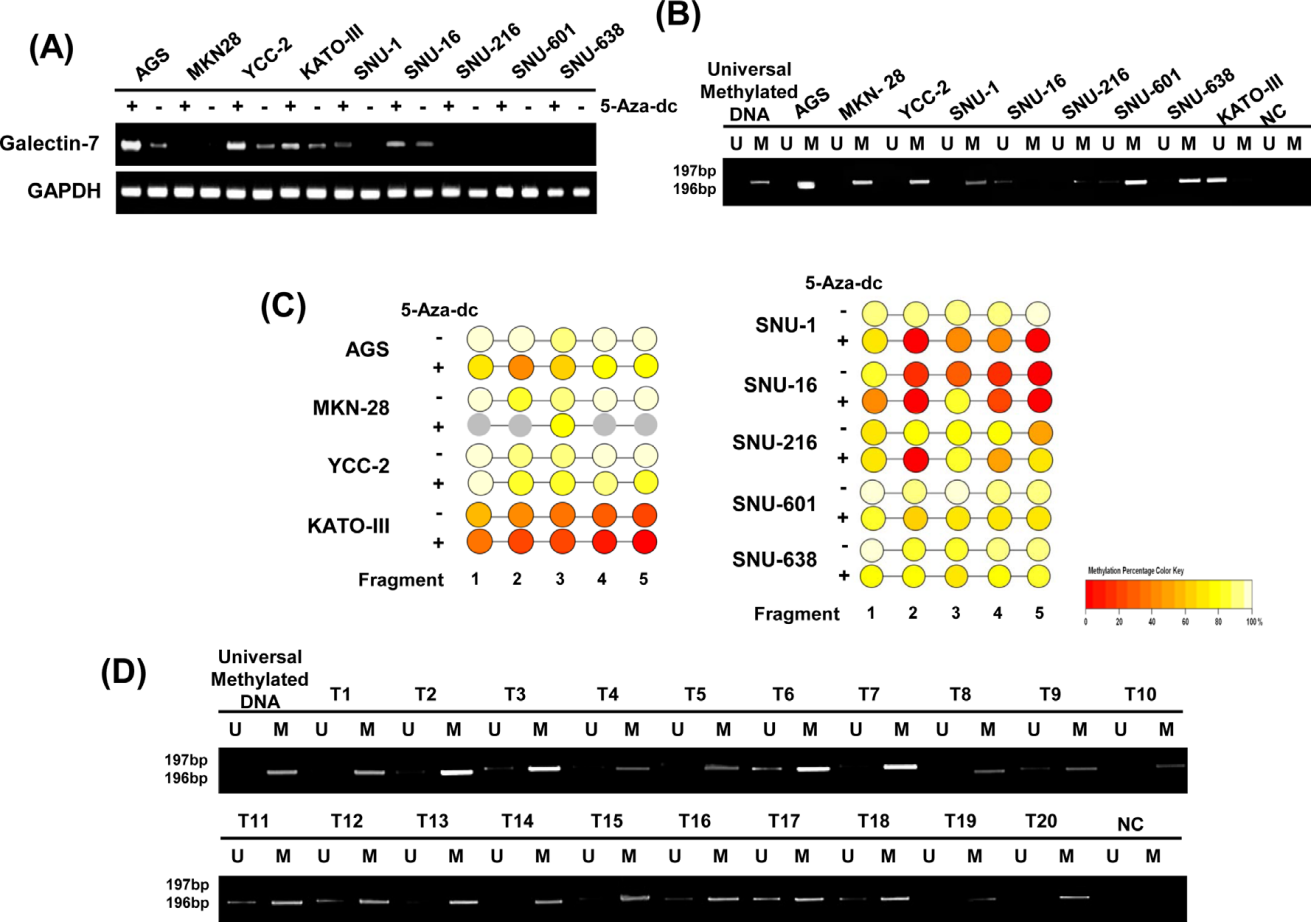
Galectin-7 was hypermethylated in gastric cancer cells and in malignant tissues of gastric cancer patients (A) mRNA expression of galectin-7 in nine gastric cancer cell lines following treatment with 50 nmol/L 5-Aza-dC. GAPDH was used as a loading control. (B) MSP analysis of the galectin-7 gene in 9 gastric cancer cell lines (C) and 20 gastric cancer patients. The methylated (M) and unmethylated (U) DNA was amplified using primers specific for each methylation status. Universal methylated DNA was used as a positive loading control. (D) Epityper Comparison of methylation patterns in nine gastric cancer cell lines (AGS, MKN28, KATOIII, YCC-2, SNU-1, SNU-16, SNU-216, SNU-601, SNU-638) after treatment with 50 nmol/L 5-Aza-dC.

To analyze the regulatory mechanism of galectin-7 expression we studied DNA demethylation by 5-aza-dC treatment. Galectin-7 mRNA expression was greatly increased in five cell lines, AGS, YCC-2, SNU-1, SNU-601 and SNU-638, following 5-aza-dC treatment (Figure 5A), suggesting that DNA methylation suppresses galectin-7 gene expression in these gastric cancer cell lines.

Next, we quantitatively analyzed the methylation status of galectin-7 in nine gastric cancer cell lines. As shown in Supplementary figure 1A, there are three CpG islands between −3000 and +2550 of the *LGALS7* gene. Among these CpG islands, we chose a 1.6-kb region (+912 to +2550) including the CpG sites at +1450 and +1800, and analyzed five amplicons as shown in Supplementary figure 1B, such that 43 CpG sites per sample were analyzed. Primers were designed using EpiDesigner software (http://www.epidesigner.com), and the sequences are shown in Supplementary figure 1C. According to Figure 5B and Supplementary figure 2, we detected more than 80% methylation in the CpG islands at +1566 bp of exon 2 of galectin-7 in seven of the nine gastric cell lines tested. In contrast, the methylation status in KATOIII and SNU16 cell lines was lower than 40%, consistent with previous results. To confirm whether down-regulation of galectin-7 in gastric cancer cell lines depends on promoter methylation, we treated nine gastric cancer cell lines with 5-aza-dC and quantitatively monitored the change in methylation status by the EpiTYPER^™^ assay (Figure 5 B and supplementary figure 2). After treatment with 5-Aza-dC, the CpG island at +1566 bp of exon 2 was demethylated about 20% in all cell lines except SNU216 (Figure 5 B and supplementary figure 2). These data indicate that the region of CpG islands at +1566 bp in exon 2 of galectin-7 is highly methylated in gastric cancer.

### 5-Aza-dC treatment increased mRNA expression of galectin-7 in 26 cancer cell lines from five different organ origins

**Figure 6:**
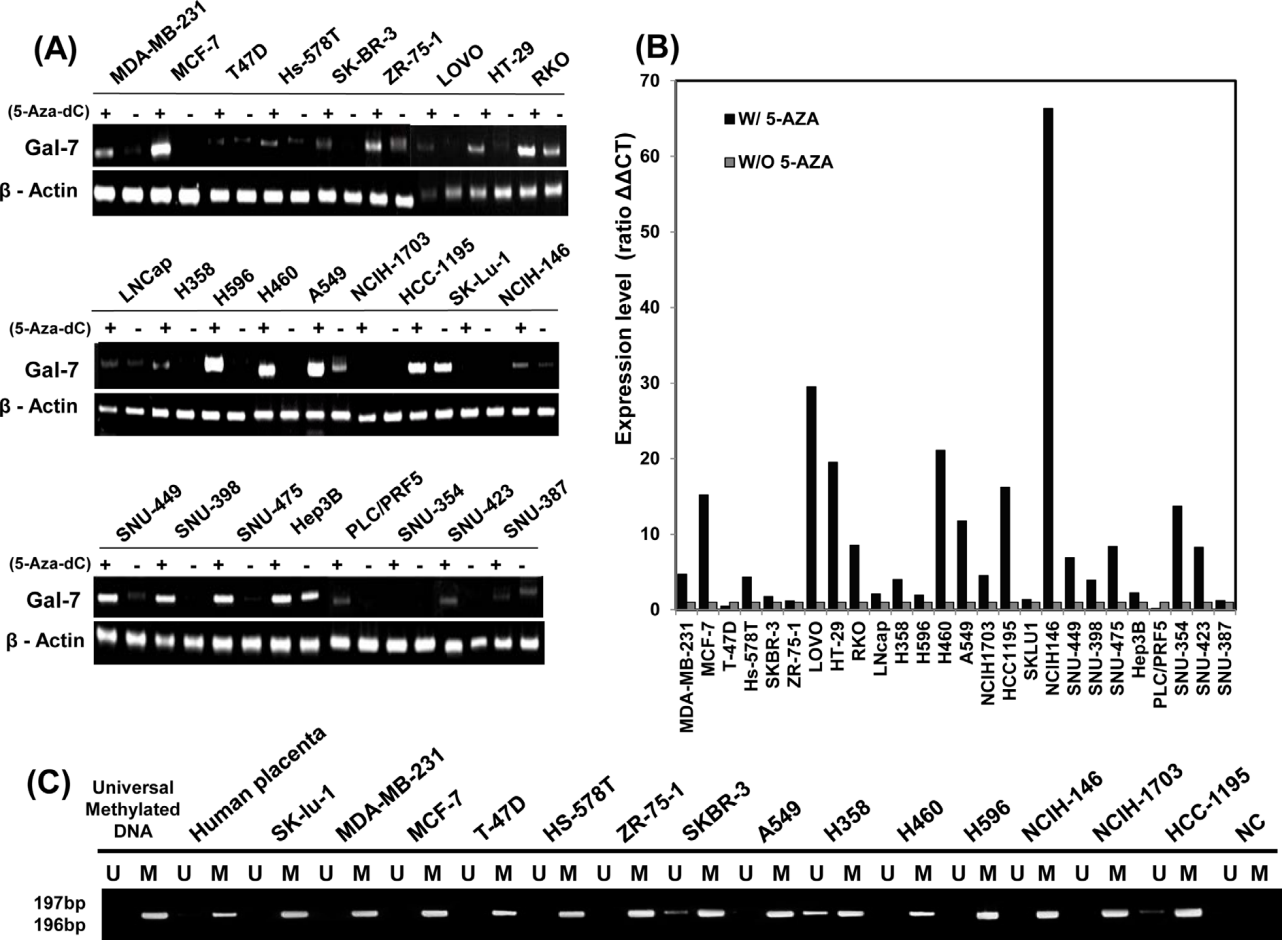
Up-regulation of galectin-7 expression in various cancer cell lines (A-B) mRNA expression of galectin-7 in 26 cancer cell lines detected by RT-PCR (A) and RT-qPCR (B). β-actin was used as a loading control. (C) MSP analysis of the galectin-7 gene in 14 cancer cell lines. The methylated (M) and unmethylated (U) DNA was amplified using primers specific for each methylation status. Universal methylated DNA was used as a positive loading control.

To further determine the importance of DNA methylation in the regulation of galectin-7 gene expression in various tumor cells, we treated 26 cancer cell lines from five different organ origins with 50 nmol/L 5-Aza-dC and performed RT-PCR analysis (Figure 6A) and real-time RT-PCR analysis (Figure 6B). Galectin-7 expression levels in various tumor cell lines markedly increased following treatment with 5-aza-dC as listed in Supplementary table 1. From the 26 cell lines we choose 6 breast cancer cell line and 8 lung cancer cell lines for MS-PCR analysis. As shown in Figure 6C, the test region of galectin-7 was hypermethylated in these cancer cell lines. Taken together, our results indicate that galectin-7 expression is regulated by DNA methylation in various cancer cell lines from different organ origins, including gastric cancer cell lines.

## DISCUSSION

Gastric cancer is difficult to cure unless it is found at an early stage before it has begun to spread. Unfortunately, because early stomach cancer causes few symptoms, the disease is usually advanced when the diagnosis is made [16]. Treatment for stomach cancer may include surgery, chemotherapy, and/or radiation therapy. Even patients who present in the most favorable condition and undergo curative surgical resection often die of recurrent disease [17]. Therefore, it is extremely important to discover a biomarker to determine the condition or stage of gastric cancer in order to improve patient survival. In this study, we suggest that galectin-7 functions as a gastric cancer suppressor. We show that the expression of galectin-7 was lower in malignant tissues than normal gastric tissues, and over-expression of galectin-7 suppressed the proliferation, migration, and invasion of gastric cancer cells. However, the role of galectin-7 in cancer tumorigenesis remains unclear because there are several conflicting reports of galectin-7 function. At first, galectin-7 was considered a pro-apoptotic factor via p53 [6-7, 10-11, 18]. However, its role in promoting tumor malignancy was also described in several reports [19-21], and galectin-7 expression was linked to increased metastatic phenotype in breast cancers [15] and migration of corneal epithelial cells [22]. The increase in tumor malignancy and metastatic phenotype were attributed to the induction of MMP-9 expression by galectin-7 [12, 23-24]. Therefore, we aimed to elucidate the mechanism by which galectin-7 suppresses gastric cancer proliferation and invasion.

Our data suggest that the expression of galectin-7 in gastric cancer is regulated by DNA hypermethylation. Aberrant DNA methylation has been shown to be involved in gastric tumourigenesis, suggesting that it may be a useful clinical biomarker for the disease [16, 25-27]. DNA methylation is an epigenetic mechanism of transcriptional regulation, and its involvement in cancer is attributed to inappropriate silencing of tumor suppressor genes or loss of oncogene repression [28-29]. Gastric cancer studies have shown that more than 100 genes exhibit aberrant DNA methylation, including significant changes in methylation of a number of genes commonly implicated in tumorigenesis, such as *MLH1*, *p16*, *CHFR*, and *RUNX3* [16, 25-27, 30], and DNA methylation of these genes might be useful for gastric cancer screening, prognostication, and treatment prediction. Here, we demonstrated diminished expression of galectin-7 in malignant tissues relative to matched normal tissues in 70% of gastric cancer patients, and also detected DNA methylation of the galectin-7 gene in malignant tissues of other gastric cancer patients. The observed increase in expression of galectin-7 in gastric cancer cell lines following 5-aza-dC treatment strongly supports the involvement of epigenetic regulation in cancer cell-specific expression. Interestingly, the hypermethylation region in galectin-7 is a predicted p53 binding site, suggesting an association with p53 function (Supplementary figure 3). Galectin-7 is considered a p53 inducible gene [10] and we also detected increased expression of gamectin-7 by DNA damaging agents such as Adriamycin (data not shown). It is possible that galectin-7 expression is less affected by p53 when under hypermethylation. However, the correlation between galectin-7 hypermethylation and transcriptional regulation by p53 requires further study. We also detected DNA hypermethylation in 24 among 26 cancer cell lines from five different organ origins. Therefore, we propose that the expression of galectin-7 is critically regulated by DNA hypermethylation and might play a role in gastric cancer tumorigenesis and as a prognostic marker of gastric cancer.

In conclusion, given the importance of early detection for improving gastric cancer survival outcomes, the results of this study indicate that DNA methylation of galectin-7 is a promising candidate biomarker for application in gastric cancer. However, it is necessary to analyze a larger number of gastric cancer patient samples to confirm these findings. Moreover, the role of galectin-7 in gastric tumorigenesis should be further examined in future studies.

## METHODS

### Cell culture

The following cell lines were obtained from American Type Culture Collection (Manassas, VA) and the Korea Cell Line Bank: six breast cancer cell lines (MDAMB231, MCF-7, T47D, Hs-578T, SK-BR-3, ZR-75-1), three colon cancer cell lines (LOVO, HT-29, RKO), eight lung cancer cell lines (A549, H358, H460, H596, NCIH-146, NCIH-1703, HCC1195, SK-Lu-1), eight liver cancer cell lines (SNU-449, SNU-398, SNU-475, Hep3B, PLC/PRF5, SNU-354, SNU-423, SNU-387), one prostate cancer cell line (LNCap), and seven gastric cancer cell lines (AGS, MKN28, KATOIII, YCC-2, SNU-1, SNU-16, SNU-216, SNU-601, SNU-638). The ATCC and KCLB authenticate the phenotypes of these cell lines. The cells were treated with 50 nmol/L 5-aza-2V-deoxycytidine (5-Aza-CdR; Sigma Chemical Company, St. Louis, MO, USA) for 3 days in three independent experiments.

### Tissue samples from gastric cancer patients

Two pairs of 2-mm tissue biopsy specimens were obtained from 20 patients with gastric adenocarcinoma during diagnostic endoscopy and endoscopic submucosal dissection. All experiments were approved by the Institutional Review Board of the National Cancer Center, approval number NCCNSH 03-024. All participants provided written informed consent for the use of tissues for comprehensive experiments on gastric cancer. For immunohistochemical analysis, core tissue biopsy specimens (2-mm diameter) were obtained from Superbiochips Laboratories (Seoul, Korea). Immunohistochemical analysis was performed as described previously [31-32] and anti-galectin-7 antibody was kindly provided by Moon-jae Cho (Jeju University).

### Generation of galectin-7 over-expressing gastric cancer xenografts in mice

All animal experiments were approved by the Yonsei University Medical School animal care and use committee (2012-0132) and were performed in specific pathogen-free facilities under conditions in accordance with the Guidelines for the Care and Use of Laboratory Animals of YUMS. The preparation of xenografted mice has been described in a previous study [31]. Galectin-7 overexpression vector pQE-9-Galectin-7, kindly provided by Moon-jae Cho (Jeju University), was transiently transfected into cells using Lipofectamine 2000 reagent (Invitrogen) according to the manufacturer's instructions.

### Western blot analysis

Western blot analysis was carried out using methodology from a previous study [32]. The primary antibodies for galectin-7 and β-actin, and HRP-conjugated goat anti-mouse or goat anti-rabbit IgGs were purchased from Santa Cruz Biotechnology and the signals were detected using an enhanced chemiluminescence (ECL) plus kit (Amersham Life Science) and a LAS-3000 (Fujifilm) detector according to the manufacturer's directions.

### RNA isolation, reverse transcription-polymerase chain reaction (RT-PCR), and quantitative PCR analysis

RNA was isolated using TRIzol○RRReagent (Invitrogen) according to the manufacturer's instructions and RT-PCR was performed using a Reverse Transcription system (Promega, Madison, WI, USA). The primers used were as follows: Galectin-7, 5'-ATGTCCACCGTCCCCCACAAG-3' and 5'-TGACGCGATGATGAGCACCTC-3'; β-actin; 5'-AGCCTCGCCTTTGCCGA-3' and 5'- CTGGTGCCTGGGGCG-3'. PCR was performed following Ex-taq (TaKaRa, Shiga, JAPAN) protocols. Real-time PCR analysis of the galectin-7 gene was performed using ABI PRISM (Applied Biosystems) according to the manufacturer's instructions with the following primer sequences specific for Galectin-7: 5'-CCTTCGAGGTGCTCATCATC-3' and 5'-GAAGATCCTCACGGAGTCCA-3'. cDNA was used as a template and product was detected with the intercalating agent SYBR green I dye.

### Bisulfite conversion

The first step of methylation analysis is bisulfite conversion of genomic DNA. Bisulfite treatment converts cytosine to uracil (thymine in DNA) by bisulfite-deamination, but in 5-methyl cytosine the position 5-methyl group inhibits deamination of the amino group. Therefore, 5-methyl cytosine remains as cytosine but unmethylated cytosine is converted to uracil. One microgram of gDNA was bisulfite treated using the EZ DNA Methylation-Gold Kit according to the manufacturer's instructions with a GeneAmp PCR system 9700 (Applied Biosystems). Briefly, 1 μg of gDNA was mixed with bisulfite treatment solution and incubated at 98°C for 10 min and 64°C for 150 min. Samples were loaded onto a column, washed with ethanol solution, and incubated with desulphonation solution for 20 minutes at room temperature. After washing, the bisulfite-treated DNA was eluted and analyzed using a Nanodrop spectrophotometer (Thermo Scientific).

### Quantitative analysis of DNA methylation

For quantitative analysis of the DNA methylation status of the *LGALS7* gene, we searched the promoter sequence using the “Transcriptional Regulatory Element Database” freeware program and designed primer sequences (+912 to +2250) using SEQUENOM EpiDesigner software as shown in Supplementary figure 1A.

### Methylation-specific PCR

PCR primers were designed using the Meth primer v1.1 beta version Software to amplify a region from −3000 to +2550 within the galectin-7 (*LGALS7*) gene (Supplementary Fig 1B). This primer set was used to verify the presence of the galectin-7 gene in cancer cell lines and gastric cancer patient tissues and for sequencing of the promoter region. An unmethylated (U)-specific set of primers: TTTTAATTTTTGGTTGGATATGTTG (forward) and CTCCAAAAACCTAAATCTTAA CATC (reverse), and a methylated (M)-specific set of primers: TTTAATTTTCGGTTGGATACGTC (forward) and CTCCAAAAACCTAAATCTTAACGTC (reverse) were designed based on the positive strand of the bisulfite-converted DNA and spanned a region within the CpG island of the promoter. The experiments were performed as described previously [33].

### Statistical Analysis

Data for cell proliferation, cell migration, and invasion assay were obtained from at least three independent experiments and are presented as mean ± SD, unless otherwise indicated. Statistical analysis was performed using Student's t-test. The Χ^2^ test was conducted to determine the significance of the difference between covariates. Survival durations were calculated by the Kaplan-Meier method. The log-rank test was used to compare cumulative survival among the patient groups. Statistical significance was set at *p*<0.05 throughout the study. The Med-Calc software program (version 11.4; MedCalc Software, Mariakerke, Belgium) was used for all analyses.

## Supplementary Figures


