# Supplementary material for: Galectin-7 is epigenetically-regulated tumor suppressor in gastric cancer
Source: Oncotarget. 2013 Aug 20;4(9):1461–71. doi: 10.18632/oncotarget.1219 (PMC3824540; doi:10.18632/oncotarget.1219)
Supplement: Supplementary file 1 [file oncotarget-04-1461-s001.pdf]

Galectin-7 is epigenetically-regulated tumor suppressor in gastric cancer - Kim et al

(A)

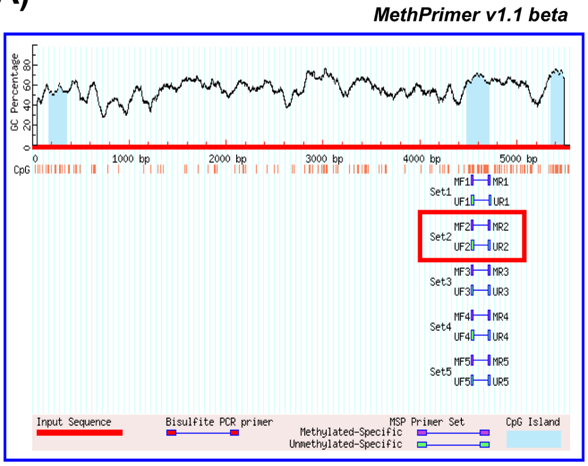

196bp	Left M primer	TTTAATTTTCGGTTGGATACGTC	23mer
	Right M primer	CTCCAAAAACCTAAATCTTAACGTC	25mer
197bp	Left M primer	TTTTAATTTTTGGTTGGATATGTTG	25mer
	Right M primer	CTCCAAAAACCTAAATCTTAACATC	25mer

(B)

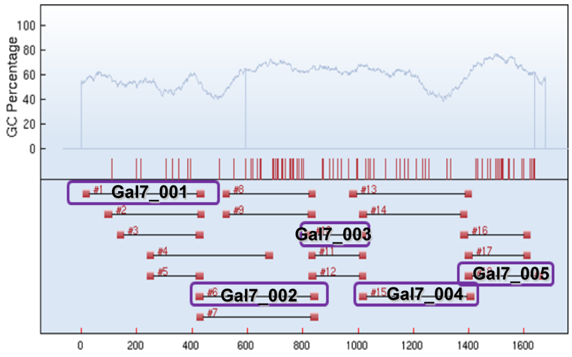

(C)

Primer ID	Sequence (5'->3')
Gal7_001_10F	aggaagagagTTTTGTAGAAGTTGAGGGTTTTTTG
Gal7_001_T7R	cagtaatacgactcactatagggagaaggctAACTCACCCTATAATCCCAACACTC
Gal7_002_10F	aggaagagagTTTAGAGTGTTGGGATTATAGGGTG
Gal7_002_T7R	cagtaatacgactcactatagggagaaggctACCAATAACCTAACTTTACCCAACC
Gal7_003_10F	aggaagagagGATTTAGGTTTTTGGAGTAGAGGGT
Gal7_003_T7R	cagtaatacgactcactatagggagaaggctCCCACCCCTTAAACACTTAACTAAT
Gal7_004_10F	aggaagagagATTAGTTAAGTGTTTAAGGGGTGGG
Gal7_004_T7R	cagtaatacgactcactatagggagaaggctATAACCCTAAAAAAATCACTCATCC
Gal7_005_10F	aggaagagagGTTTAGAGGGGATGAGTGATTTTTT
Gal7_005_T7R	cagtaatacgactcactatagggagaaggctTCATCTCAATAAATAAAACACAAACACA

Supplementary Figure 1: High-throughput analysis of informative CpG sites for the galectin-7 gene. (A) Schematic view of the *LGALS7* gene CpG region from −3000 to +2550 of gene. The positions of primers for MSP were established using the methprimer program. (B-C) Size and CpG sites of the amplicons in the *LGALS7* gene. (B) Based on CpG sites, primers were designed for *in vitro* transcription as a reverse primer with a T7 promoter tag and a forward primer with a 10-mer tag sequence as a balance.

(A)

Cell lines \ 5-Aza	(-)	(+)
AGS	92 %	64 %
MKN28	89 %	76 %
KATOIII	37 %	17 %
YCC-2	92 %	83 %
SNU1	89 %	27 %
SNU16	26 %	32 %
SNU216	70 %	56 %
SNU601	88 %	68 %
SNU638	85 %	77 %

(B)

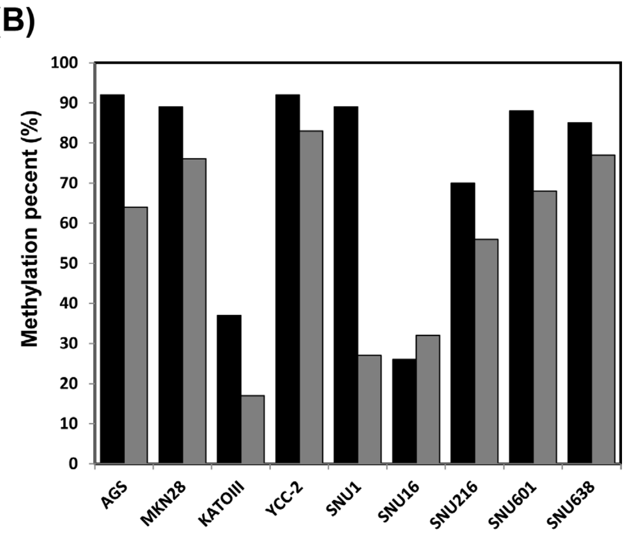

Supplementary Figure 2: Galectin-7 was hypermethylated in gastric cancer cells. (A-B) Percentage values and histogram from Epityper Comparison of methylation patterns in nine gastric cancer cell lines (AGS, MKN28, KATOIII, YCC-2, SNU-1, SNU-16, SNU-216, SNU-601, SNU-638) after treatment with 50 nmol/L 5-Aza-dC.

(A)

Transcription Factor	TF BS
OCT1	-2665
Sox-9	-1759
CP-2	-1328
CP-2	-1296
AP-1	-545
NF-1	-97
p53	-41
SREBP-1a / b / c	503
HSF1	1022
ADR1	1231
ELK1	1264
HSF1	1422
p53	1566

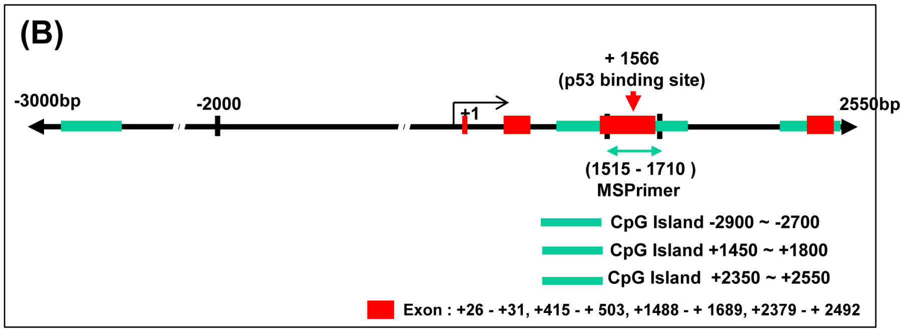

Supplementary Figure 3: CpG methylation status of the *LGALS7* gene. (A) Transcription factor binding factors. (B) Schematic view of the *LGALS7* gene from -3000 to +2550, showing the CpG region of exon 2 (+1488 – +1689), MS-PCR primer region, and p53 binding site.

Cell line	Organ	PCR	qRT-PCR	Cell line	Organ	PCR	qRT-PCR
MDA-MB-231	Breast	I	I	SNU-449	Liver	I	I
MCF-7		I	I	SNU-398		I	I
T-47D		I	I	SNU-475		I	I
Hs-578T		I	I	Hep3B		I	I
SK-BR-3		I	I	PLC/PRF5		I	I
ZR-75-1		I	I	SNU-354		-	I
A549	Lung	I	I	SNU-423		I	I
H358		I	I	SNU-387		D	D
H460		I	I	LNCap	Prostate	I	I
H596		I	I	LOVO	Colon	I	I
NCIH-146		I	I	HT29		I	I
NCIH1703		D	D	RKO		I	I
HCC1195		I	I				
SK-lu-1		I	I				

I=increase, D= decrease

Supplementary Figure 4: Galectin-7 expression in 26 cancer cell lines following 5-Aza-dC treatment.
